# Evaluation of Object Surface Edge Profiles Detected with a 2-D Laser Scanning Sensor

**DOI:** 10.3390/s18114060

**Published:** 2018-11-21

**Authors:** Tingting Yan, Xiaochan Wang, Heping Zhu, Peter Ling

**Affiliations:** 1 College of Engineering, Nanjing Agricultural University, Nanjing 210031, China; tingting.yan@ars.usda.gov; 2 USDA-ARS Application Technology Research Unit, Wooster, OH 44691, USA; 3 Department of Food, Agricultural and Biological Engineering, The Ohio State University, Wooster, OH 43210, USA; ling.23@osu.edu

**Keywords:** automation, surface contour detection, greenhouse crop, pesticide spray equipment, variable rate

## Abstract

Canopy edge profile detection is a critical component of plant recognition in variable-rate spray control systems. The accuracy of a high-speed 270° radial laser sensor was evaluated in detecting the surface edge profiles of six complex-shaped objects. These objects were toy balls with a pink smooth surface, light brown rectangular cardboard boxes, black and red texture surfaced basketballs, white smooth cylinders, and two different sized artificial plants. Evaluations included reconstructed three-dimensional (3-D) images for the object surfaces with the data acquired from the laser sensor at four different detection heights (0.25, 0.50, 0.75, and 1.00 m) above each object, five sensor travel speeds (1.6, 2.4, 3.2, 4.0, and 4.8 km h^−1^), and 8 to 15 horizontal distances to the sensor ranging from 0 to 3.5 m. Edge profiles of the six objects detected with the laser sensor were compared with images taken with a digital camera. The edge similarity score (ESS) was significantly affected by the horizontal distances of the objects, and the influence became weaker when the objects were placed closer to each other. The detection heights and travel speeds also influenced the ESS slightly. The overall average ESS ranged from 0.38 to 0.95 for all the objects under all the test conditions, thereby providing baseline information for the integration of the laser sensor into future development of greenhouse variable-rate spray systems to improve pesticide, irrigation, and nutrition application efficiencies through watering booms.

## 1. Introduction

The timely application of pesticides is critical to protect crops from insect and disease damage [1]. However, conventional constant-rate sprayers are often reported to waste much of the spray materials, resulting in increased production costs and environmental contamination potential [2,3,4,5,6].

In order to overcome these problems, modern sprayers are expected to automatically control spray outputs precisely to match the plant configuration and foliage density in order to avoid delivering chemicals to non-target areas. In this way, high efficiency and low production costs can be achieved for pesticide spray applications with minimum environmental impact [7,8,9,10,11,12,13,14].

Earlier precision sprayer control systems only manipulate spray outputs in an on/off or intermittent manner according to the presence or absence of target plants [15,16,17]. Chemical application and crop management according to the orchard foliar volume is known as the tree row volume (TRV) concept. Chemical application would be more precise and efficient if the spray could be adjusted in real time based on the foliar volume rather than only on the presence of plants or land area.

A modern, variable-rate sprayer is developed by integrating sensor-guided technology to detect plants and determine the chemical amount to match the plant canopy volume. Thus, sensors are important factors in target structure detection to fulfill automatic variable-rate functions.

The ultrasonic sensor is one of the sensor application examples. McConnell et al. [18] propose a system to determine the volume of foliage by summing the tree measurements detected by a vertical array of transducers. Ultrasonic generators are used as foliage sensors and installed on a discrete grove sprayer to actuate spray nozzles corresponding to foliage sensed within a spray zone [19]. Giles et al. [20] develop an electronic tree canopy volume measurement system based on commercial ultrasonic range transducers. The performance of that measurement system is calibrated at different stages of leaf development in apple and peach orchards. However, the system tends to overestimate the tree width and underestimate the tree extension. Soon afterwards, an electronic measuring system for orchard tree foliage sensing and mapping is proposed for the determination of the amount and vertical distribution of sensed centroids in vertical sectors of orchard trees, and to create map of foliar volumes [21]. Recently, an experimental vertical boom spray system integrated with 20 Hz ultrasonic sensors was developed to spray liner trees in nursery production [22,23].

Laser range detection sensors have been shown to be more accurate and robust, with obvious advantages in detection of crops under field conditions compared to ultrasonic and other sensors. Instrumentations incorporated with laser sensors for crop geometry measurement have been developed and mounted on tractor platforms to adjust the spray output to compensate for the enormous crop changes in orchards for the purpose of avoiding the administration of over or under doses of pesticide [24,25,26]. Tumbo et al. [27] compare the performance of the ultrasonic and laser sensors in estimating canopy volume in citrus, and find that the laser sensor has better estimation, especially for partially-defoliated trees or small replants. Furthermore, lasers can also adapt to a wider range of temperatures and offer faster data acquisition than ultrasonic sensors. Schwartz Electro–Optics, Inc. (SEO) (Orlando, FL, USA) uses a ground-based laser sensor to detect tree foliage and produce a two dimensional profile, but canopy volumes cannot be obtained by that detection system. An airborne laser range detection system is also utilized in the canopy measurement and is validated to be significantly correlated with the measurement made with ground measurements [28,29,30].

Ultimately, a laser scanning sensor is integrated to the variable-rate spray control system of an air-assisted sprayer [31]. Another 270° radial range laser sensor is verified in the detection of complex-shaped objects, and is successfully installed in an air-assisted sprayer [32,33]. However, none of these previous studies referred to canopies as potentially small and dense as greenhouse grown plants.

In greenhouse spray application, handgun equipment and spray booms are common choices, while the spray booms are demonstrated to work better in efficiency and reducing deposition variations at different locations in canopy than handgun equipment [34,35,36].

Targets are placed under the spray booms in greenhouses implemented with boom spray applications. A simple, inexpensive laser scanning sensor for indoor use may be suitable for variable-rate applications if the sensor could be mounted on the spray boom with its scanning plane facing toward the ground [37].

As laser sensors play an important role in plant volume estimation for precise spray, they are of significant importance to validate the detection accuracy of the laser sensor before being integrated into real spray systems. However, current applications of laser sensors on field sprayers are mostly based on the ability of the laser sensor from the side view to detect large and tall plants individually, which is not adaptable for the horizontal spray booms used in greenhouse to treat the pot-grown plants placed on the ground. In previous research [37], the accuracy of the laser sensor in detecting complex-shaped objects along X (width or horizontal distance) direction, Y (length or sensor speed) direction and Z (height or vertical) direction is validated. The dimensions of all the objects detected by the laser sensor matched with the actual dimensions in Y and Z directions very well under indoor conditions. The highest root mean square error (RMSE) and coefficient of variation (CV) are 83 mm and 50.9% in the horizontal direction, 41 mm and 15.2% in the travel direction, and 16 mm and 14.0% in the height direction, respectively. However, the canopy information of the plant could not be fully represented by the measurement along those three coordinates alone. Edges are of fundamental importance to characterize boundaries and preserve the important structural properties in an image, as they correspond to discontinuities in the physical and geometrical properties of scene objects [38,39]. Thus, edge detection is critical for object recognition [40,41] to compensate for the shortage of the three-coordinate-measurement of targets in previous research.

Numerous edge detectors have been reported over the past 50 years. Computing color gradient magnitudes followed by non-maximal suppression is a traditional approach for edge detection [42,43]. The detection of intensity or color gradients is also developed for edge detection of images with certain orientations [44,45,46]. However, they perform poorly for images with blurred and noisy edges. To solve this problem, sophisticated operators such as linear operators have been developed with better immunity to noise for more accurate edge detection. Shen and Castan [47] propose that the symmetric exponential filter of an infinite size (ISEF) is optimal in detecting mono- and multi-edge. A structured learning approach is also applied to edge detection, and shows surprising computationally efficiency [48]. A structure-from-motion task and empirical receiver operating characteristic (ROC) curves are applied in a framework to evaluate the edge detector [49,50].

With evidence of the dimension measurement accuracy of the laser sensor in previous research, the goal of this research was to evaluate the edge profile detection accuracy of the indoor use 270° radial range laser scanning sensor before its integration to the greenhouse variable-rate spray system. The primary objectives were (1) using the laser sensor to scan the complex-shaped objects at different horizontal distances, sensor speeds, and detection heights under indoor conditions; (2) analyzing the detection resolution of the laser sensor; (3) reconstructing the pseudo-color images mapping the 3-D object surface and detecting the edge profiles of the paired images from the laser sensor and the digital camera; (4) validating the edge detection accuracy of the laser sensor with the edge similarity score (ESS) of pared images from the laser sensor and camera.

## 2. Materials and Methods

### 2.1. Laser Sensor and Data Acquisition

The laser-scanning sensor used for tests was an indoor use sensor with 10 m detection range (Model UST-10LX, Hokuyo Automatic Co., Ltd., Osaka, Japan). Its light weight (130 g) and small size (50 × 50 × 70 mm) provided flexibilities for installation on moving frames. The sensor transmitted 1080 laser data points in the 270° full radial range at 0.25° angular resolution in a 25 ms scanning cycle. The 270° fan-shaped detection plane of the sensor was oriented perpendicular to both the floor and the sensor travel direction, which enabled the laser sensor to continuously scan the bilateral plants surfaces (540 points on each side). The laser transmitter inside the sensor was driven by a precision step motor rotated evenly at a resolution of 0.25° with the laser wavelength of 905 nm. The object position and distance to the sensor could be calculated from the reflect signals and rotation angles of their transmitted laser beams. The laser sensor was connected to an embedded computer (MXE-1005, Fanless Embedded Computer, ADLINK Technology Inc., Taiwan) via an Ethernet interface connection. A specially-designed program was written in VC++ language and was installed in the embedded computer to filter the uninterested data from the laser sensor, reconstruct 3-D pseudo-color object surface with a format of gray-scale values, calculate the desired amount of spray needed for each nozzle operation, and operate the sprayer. The program was very similar to the program reported by Liu and Zhu [32]. The contours of the images for the detected objects were also constructed by the morphological operator and edge detection method [31,32].

### 2.2. Laboratory Tests

**Figure 1:**
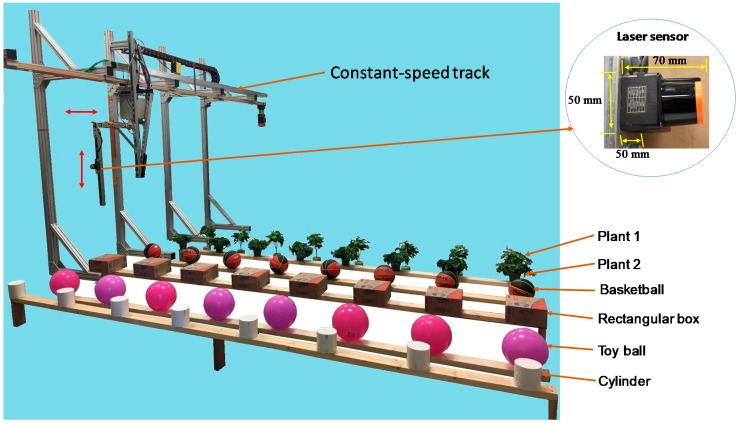
The 270° radial range laser sensor mounted on a constant-speed track to detect six rows of different objects (artificial plant 1, artificial plant 2, basketball, rectangular box, toy ball, and cylinder).

The accuracy of the laser sensor in detecting the edge profiles of complex-shaped objects was validated in a 5 m wide, 8 m long and 5.5 m high indoor area with 100 lux illuminance light intensity. The ambient temperature was 23 °C and relative humidity was 15%. The sensor was attached on a height-adjustable bracket which was mounted to a constant-speed track, and the 270° fan-shaped detection plane of the sensor faced vertically toward the ground. The sensor height could be adjusted in a range from 0 to 1.5 m above targets. The detection targets were four regularly-shaped objects and two artificial plants (Figure 1). The regularly-shaped objects were toy balls with a pink smooth surface, light brown rectangular cardboard boxes, black and red texture surfaced basketballs, white smooth cylinders (hereafter referred to as “toy ball”, “rectangular box”, “basketball”, and “cylinder”, respectively). The two artificial plants were named “Plant 1” and “Plant 2”. Real greenhouse plants were not chosen for the test to avoid dimension changes over the experiment period.

The diameters of toy balls and basketballs were 233 and 183 mm, respectively. The rectangular boxes were 231 mm wide, 225 mm long, and 115 mm high. The cylinders were 108 mm high and 118 mm in diameter. The dimensions of artificial plants were 231 mm wide, 232 mm long, and 325 mm high for plants 1, and 254 mm wide, 277 mm long and 179 mm high for plants 2, respectively. The edge profiles of all the objects constructed from images by digital camera were used for standard comparisons with the profiles constructed from images by the laser sensor.

During the test, eight of the same objects were classified into a row, evenly. Six types of objects were placed in six rows, parallel to each other. The first object in one row was located directly under the sensor travel path, and the last object was 3.5 m from the first object. This test consisted of two parts, Part 1 and Part 2; the variables in each part were listed in Table 1. As the nozzle space on greenhouse spray booms was usually 0.5 m, the interval distance between objects was set as 0.5 m in Part 1. With this setting, the object was right below the nozzle on the greenhouse spray boom. Correspondingly, the interval spacing between two objects in the same row was 0.27, 0.31, 0.27, 0.38, 0.27 and 0.25 m for toy ball, basketball, rectangular box, cylinder, artificial plant 1, and plant 2, respectively. To simulate a denser crop, the interval distance was shortened to 0.25 m. Similarly, the spacing between two objects in the same row became 0.02, 0.06, 0.02, 0.13, 0.02 and 0 m for toy ball, basketball, rectangular box, cylinder, artificial plant 1, and plant 2, respectively. In this research, the vertical distance between the laser sensor and the top of objects was defined as the detection height.

### 2.3. Data Analysis

#### 2.3.1. Detection Resolution of the Laser Sensor

Because the laser sensor was mounted with its fan-shaped detection plane along the horizontal distance direction and scanned the objects from one side to the other side, detection resolution (DR) was analyzed along the horizontal direction (DRH) and the sensor travel direction (DRS). In the sensor travel direction, as the detection cycle time of the laser sensor was 25 ms, DRS was affected by the sensor travel speeds; the faster the speed, the lower the DRS.

**Figure 2:**
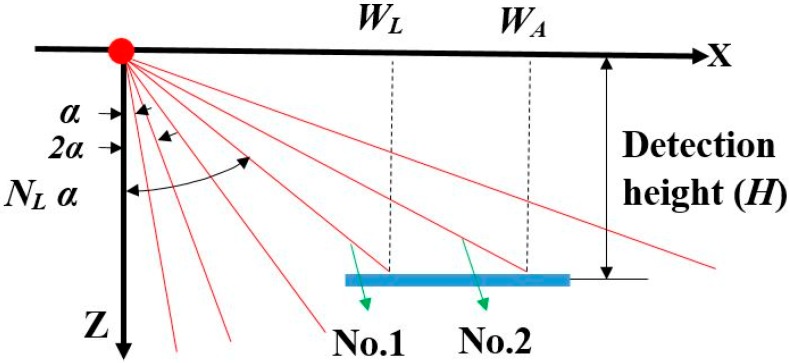
Geometry analysis of the laser beam points transmitted on the object surface on one side of the sensor.

As the angular resolution of the laser sensor was 0.25°, the distance between two adjacent laser data points varied with the distance away from the sensor, which was the same to that using laser sensors to detect vertical objects [32]. Hence, DRH varied with the dissimilar horizontal distances and detection heights. For a flat surface parallel to the ground on one side of the sensor (taking the minus side as an example), red solid lines shown in Figure 2 represented the laser flux. The blue bold solid line represented the object surface. It was assumed two adjacent laser beams, No. 1 and No. 2, transmitted on the object surface, whose horizontal distances were *W_L_* and *W_A_*, respectively. DRH could be calculated with the Equation (1) based on the geometry analysis (Figure 2).

(1) $$\mathrm{DRH}=\left|W_{A}-W_{L}\right|=1000\times H\times \left\{\tan\left[\left(N_{L}+1\right)\times \frac{\pi \alpha }{180}\right]-\tan\left(N_{L}\times \frac{\pi \alpha }{180}\right)\right\},$$

where *H* is the detection height, α is the sensor angular resolution (0.25°), *N_L_* is the numbers of points that reach the surface at width *W_L_* (take the minus side as an example).

The following equations were used to calculate the values of *N_L_*:

(2) $$N_{L}=1+Int\left[\frac{\arctan\left(\frac{W_{L}}{H}\right)}{\frac{\pi \alpha }{180}}\right].$$

#### 2.3.2. Paired Image Similarity Evaluation

In this test, edge similarity score (ESS), based on the distance fields computation, was used to quantify the similarity of paired images from the digital camera and the laser sensor. Distance fields were widely used in shape representation [51,52], object simplification [53], remeshing [54], sculpting [55], swept volume computation [56], path planning and navigation [57,58], collision and proximity computations [59,60], etc. Different algorithms have been proposed to compute the distance fields of geometric models in 2-D and volumetric models in 3-D. Sud and Manocha [61] presented a fast algorithm (DiFi) to compute a discretized distance field of complex objects. For the image similarity evaluation in the test, the absolute correlation coefficient between two edge distance fields of the paired images from the laser sensor and the digital camera was used to calculate ESS as Equation (3) [32]:

(3) $$\mathrm{ESS}=abs\left(\frac{\sum_{j=1}^{n}\left(\alpha_{j}-\bar{\alpha }\right)\left(\beta_{j}-\bar{\beta }\right)}{\sqrt{\sum_{j=1}^{n}{\left(\alpha_{j}-\bar{\alpha }\right)}^{2}}\sqrt{\sum_{j=1}^{n}{\left(\beta_{j}-\bar{\beta }\right)}^{2}}}\right),$$

where *α_j_* and *β_j_* (*j* = 1, 2, ⋯, *n*) are pixel points at the edge profiles of paired images obtained from the laser sensor and camera respectively, and $\bar{\alpha }$ and $\bar{\beta }$ are the mean values of *α_j_* and *β_j_*. ESS ranges from 0 to 1:0 means there is no similarity between the edge profiles of the paired images, while 1 means the two profiles are exactly same. Thus, greater ESS presented higher similarity of the paired images.

## 3. Results and Discussion

### 3.1. Detection Resolution of the Laser Sensor

For the sensor travel direction, DRS was proportional to the speeds because of the 25 ms scanning cycle time. The DRS was 11, 16, 22, 27 and 33 mm at speeds of 1.6, 2.4, 3.2, 4.0 and 4.8 km h^−1^, respectively. Under ideal conditions, there would be, at most, a single laser beam that could reach the objects when their dimensions in the travel direction were smaller than these resolutions.

**Figure 3:**
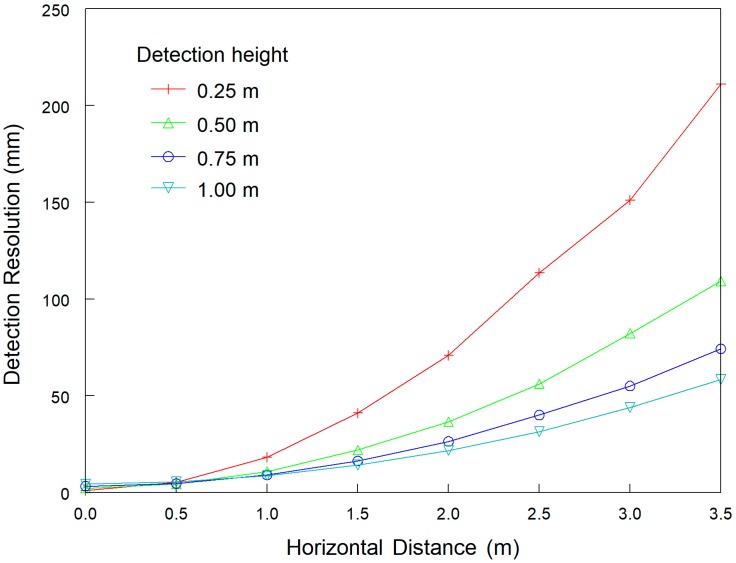
Calculated detection resolution of the laser sensor along the horizontal direction (DRH) with Equation (1) for different horizontal distances and detection heights.

The DRH was significantly affected by the sensor detection heights and horizontal distances. The horizontal distance influenced DRH more significantly when the detection was conducted at a relatively low detection height (Figure 3). DRH increased as the object horizontal distance increased at all detection heights. For example, at 0.5 m detection height, DRH was 2.5, 4.4, 10.9, 22.0, 36.6, 56.1, 82.2 and 109.2 mm when the horizontal distance was 0, 0.5, 1.0, 1.5, 2.0, 2.5, 3.0 and 3.5 m, respectively. The DRH at lower detection height increased more rapidly with the increase of horizontal distance than DRH at higher detection height. The DRH did not have a constant increase or decrease trend with increase of the detection height. For example, at 0 m horizontal distance, DRH increased from 1.1 to 4.4 mm as the detection height increased from 0.25 to 1.0 m. However, at the 3.5 m horizontal distance, DRH was 211.0, 109.2, 74.2 and 58.3 mm for the detection height of 0.25, 0.5, 0.75 and 1.0 m, respectively. These results revealed that relatively higher detection height could weaken the negative influence of the horizontal distances on DRH and increase the detection accuracy.

### 3.2. Object Outline Profile Similarity

**Figure 4:**
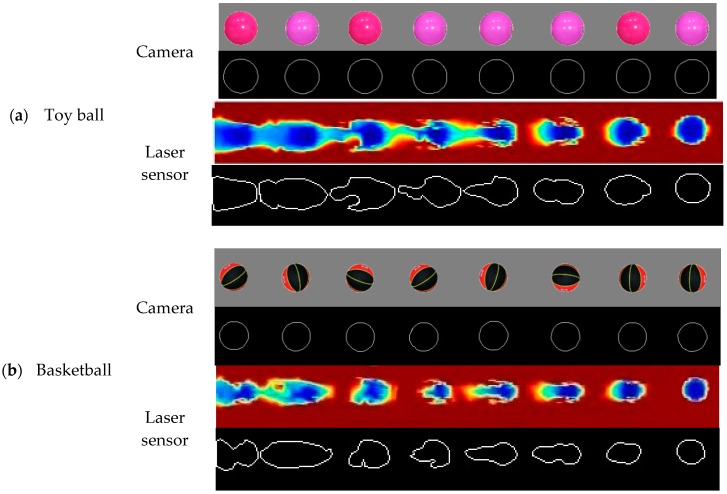

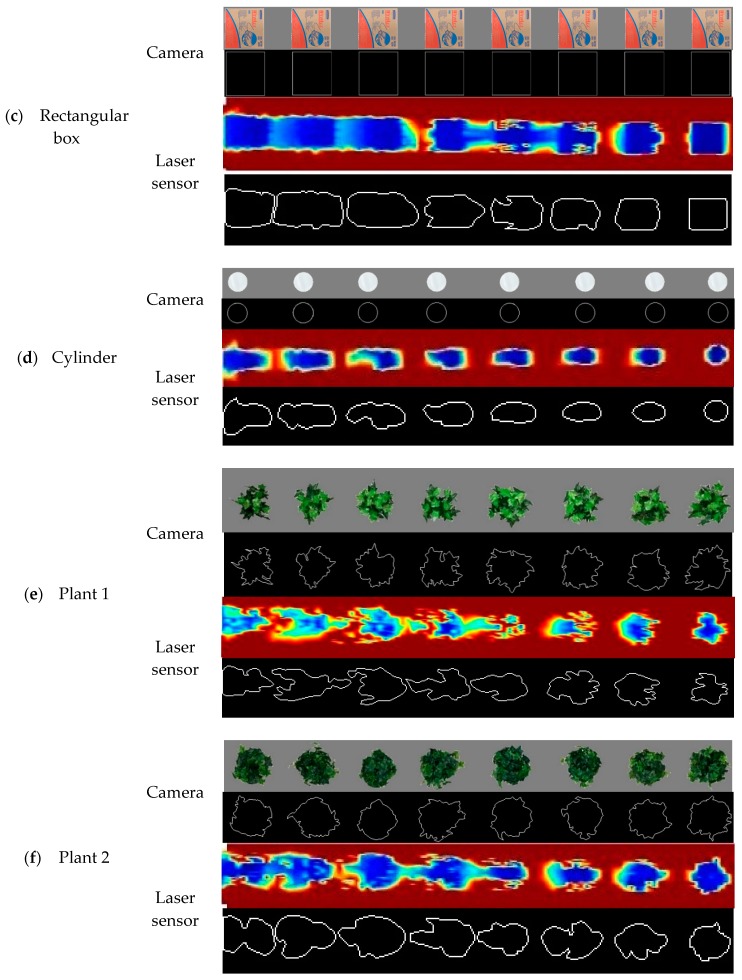
Processing edge similarity score (ESS) for paired images of eight 0.5 m evenly spaced objects obtained from the camera (upper) and laser sensor (lower): (**a**) toy ball, (**b**) basketball, (**c**) rectangular box, (**d**) cylinder, (**e**) artificial plant 1, and (**f**) artificial plant 2. The reconstructed images from the laser sensor were taken at 3.2 km h^−1^ travel speed and 0.5 m detection height. Different colors represent different vertical distances between the object surfaces and the laser sensor.

**Figure 5:**
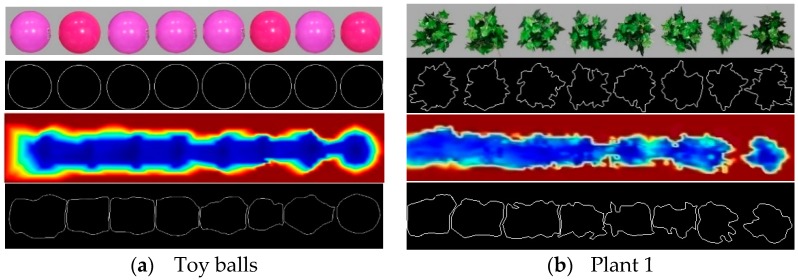
Processing ESS for paired images of eight 0.25 m evenly spaced objects obtained from the camera (upper) and laser sensor (lower): (**a**) toy ball, (**b**) artificial plant 1. The reconstructed images from the laser sensor were taken at 3.2 km h^−1^ travel speed and 0.5 m detection height. Different colors represent different vertical distances between the object surfaces and the laser sensor.

Figure 4 and Figure 5 illustrated the process of the ESS calculated from the outline profile similarity for the paired images of objects from the camera and the laser sensor. For the reconstructed pseudo-color images from the laser sensor, different colors represented different vertical distances between the sensor and the object surfaces.

#### 3.2.1. Edge Detection of Toy Balls

Travel speed had a minor influence on the measurement accuracy in the range of travel speeds evaluated. Table 2, Table 3 and Table 4 shown the mean ESS of the toy balls calculated with Equation (3) for different sensor travel speeds, detection heights, and object types, respectively. The mean ESS of eight toy balls was 0.59, 0.58, 0.56 and 0.54 at the sensor travel speed of 1.6, 2.4, 3.2, 4.0 and 4.8 km h^−1^, respectively. As mentioned above, the sensor travel speed slightly influenced the detection resolution along the travel direction. Similarly, ESS was also influenced by the sensor travel speed. For example, for the toy ball located at 0.5 m horizontal distance, the mean ESS was 0.83, 0.82, 0.81, 0.79 and 0.78 at the sensor travel speed of 1.6, 2.4, 3.2, 4.0 and 4.8 km h^−1^, respectively (Table 2).

As predicted by the calculation of DR of the laser sensor in detecting objects with different horizontal distances at different sensor detection heights, the detection accuracy was affected by both the detection height and horizontal distance. The horizontal distance influenced the detection more significantly. For the eight toy balls at detection heights 0.25, 0.5, 0.75 and 1.0 m, the average ESS was 0.48, 0.51, 0.61 and 0.67, respectively. For all the toy balls, the influence from the four detection heights varied with their horizontal distances. For example, the ESS ranged from 0.96 to 0.97 for the toy ball at horizontal distance of 0 m, while the range was changed from 0.54 to 0.76 at horizontal distance of 1.0 m (Table 3). That is, the detection heights slightly affected the ESS of the toy ball directly under the sensor, and this influence became greater as the horizontal distance became longer.

For the eight toy balls under all the 20 combinations of detection heights and travel speeds, the mean ESSs were 0.96, 0.80, 0.66, 0.54, 0.47, 0.38, 0.37 and 0.35 at horizontal distances of 0, 0.5, 1.0, 1.5, 2.0, 2.5, 3.0 and 3.5 m, respectively (Table 4). On the other hand, the mean ESS of the toy balls at different horizontal distances ranged from 0.98 to 0.37, from 0.97 to 0.35, from 0.96 to 0.35, from 0.96 to 0.34, and from 0.95 to 0.33 for the sensor travel speeds of 1.6, 2.4, 3.2, 4.0 and 4.8 km h^−1^ (Table 2). The mean ESS for toy balls at detection heights of 0.25, 0.5, 0.75 and 1.0 m was from 0.96 to 0.29, from 0.96 to 0.30, from 0.97 to 0.35, and from 0.96 to 0.46, respectively (Table 3). Thus, the horizontal distances influenced the edge detection significantly. All these influences from the detection heights and horizontal distances were caused by DR.

As the plant arrangement in real greenhouse could not be changed, a higher detection accuracy of the laser sensor could be obtained with a detection height greater than 1.0 m and a sensor speed lower than 1.6 km h^−1^.

#### 3.2.2. Edge Detection of Basketballs

The edge detection accuracy of basketballs was also significantly influenced by the horizontal distance, varying with the detection height, and slightly affected by the sensor speed. For the basketballs, the mean ESSs at different travel speeds and detection heights are illustrated in Table 5 and Table 6, respectively. The overall mean ESS of the basketballs across the five travel speeds and four detection heights decreased from 0.95 ± 0.61 to 0.38 ± 0.03 as the horizontal distance increased from 0 to 3.5 m. Across the different horizontal distances and detection heights, the mean ESS was 0.61, 0.59, 0.58, 0.57, and 0.56 at the sensor travel speed of 1.6, 2.4, 3.2, 4.0, and 4.8 km h^−1^, respectively. The mean ESS was 0.49, 0.53, 0.61, and 0.70 across the different speeds and horizontal distances at detection heights of 0.25, 0.5, 0.75, and 1.0 m, respectively.

Similar to the toy balls, the object horizontal distances influenced the ESS greatly. For example, the ESS ranged from 0.96 to 0.37 for the basketballs at different horizontal distances at the sensor travel speed of 3.2 km h^−1^ (Table 5). Besides, the ESS ranged from 0.36 to 0.95 for the basketballs at different horizontal distances for sensor detection height of 0.25 m, and from 0.42 to 0.95 for the sensor detection height of 1.0 m (Table 6). Hence, based on the evaluation in this research, the optimal laser scanner sensor settings would be at detection height of 1.0 m and travel speed of 1.6 km h^−1^.

#### 3.2.3. Edge Detection of Rectangular Boxes

Similar to the ball objects, the detection ability of the laser sensor in detecting rectangular box was significantly affected by the object horizontal distance and detection heights, and decreased with the sensor travel speed increasing from 1.6 to 4.8 km h^−1^. However, the overall ESS was greater than the two sized balls mentioned above, which was due to that the rectangular box surface was smoother than the ball surface. The mean ESSs of rectangular boxes at different travel speeds and detection heights are shown in Table 7 and Table 8, respectively. Across all the combinations of travel speeds and detection heights, the overall mean ESS decreased from 0.97 ± 0.01 to 0.48 ± 0.06 as the horizontal distance increased from 0 to 3.5 m (Table 4). However, the ESS slightly decreased from 0.73 to 0.67 as the sensor travel speed increased from 1.6 to 4.8 km h^−1^. The mean ESS was 0.67, 0.68, 0.72, and 0.73 at detection heights of 0.25, 0.5, 0.75 and 1.0 m, respectively.

#### 3.2.4. Edge Detection of Cylinders

Table 9 and Table 10 shown the mean ESS of the cylinders at eight horizontal distances for different travel speeds and different detection heights, respectively. The ESS was 0.61, 0.59, 0.58, 0.57 and 0.56 at the sensor travel speed of 1.6, 2.4, 3.2, 4.0 and 4.8 km h^−1^, respectively. In addition, the mean ESS was 0.52, 0.52, 0.61 and 0.66 at detection heights of 0.25, 0.5, 0.75 and 1.0 m, respectively. Similar to other regularly-shaped objects, ESS of the cylinders was considerably influenced by the horizontal distances, and slightly influenced by the travel speeds. For instance, the overall ESS across all the conditions at different horizontal distances varied from 0.31 to 0.96 (Table 4), and varied between 0.85 and 0.86 for the cylinders at horizontal distance of 0.5 m as the travel speed increased from 1.6 to 4.8 km h^−1^ (Table 9). Because of the variable DR caused by different detection heights and horizontal distances, the ESS also varied with the horizontal distance at a given detection height. For the cylinders at 0 m horizontal distance, the ESS changed slightly in the range from 0.95 to 0.96 (Table 10). However, the ESS of the cylinders at the horizontal distances greater than 0 m increased with the detection heights. For example, ESS at 1.0 m horizontal distance was 0.58, 0.63, 0.73 and 0.81 when the detection height was 0.25, 0.5, 0.75, and 1.0 m, respectively (Table 10). These results also confirmed that greater detection height could increase the edge detection accuracy of the laser sensor.

#### 3.2.5. Edge Detection of Two Artificial Plants

The mean ESS at different travel speeds and detection heights are shown in Table 11 and Table 12 for the artificial plant 1, and are shown in Table 13 and Table 14 for the artificial plant 2, respectively. Compared with the regularly-shaped objects, the ESS of the artificial plants were a little lower because of their irregular architectures. However, the overall mean ESS was still influenced considerably by the horizontal distances. The ESS for artificial plant 1 was 0.90 ± 0.02, 0.72 ± 0.02, 0.67 ± 0.04, 0.61 ± 0.03, 0.45 ± 0.08, 0.37 ± 0.09, 0.35 ± 0.09 and 0.35 ± 0.08 at the horizontal distance of 0, 0.5, 1.0, 1.5, 2.0, 2.5, 3.0 and 3.5 m, respectively. However, the overall mean ESS for plant 2 was slightly higher than that for plant 1. The ESS for artificial plant 2 was 0.94 ± 0.01, 0.81 ± 0.05, 0.78 ± 0.04, 0.64 ± 0.04, 0.48 ± 0.08, 0.44 ± 0.06, 0.41 ± 0.11 and 0.38 ± 0.09 at the horizontal distance of 0, 0.5, 1.0, 1.5, 2.0, 2.5, 3.0 and 3.5 m, respectively. This was because that the plant 2 had flatter canopy surface than the plant 1, which increased the laser sensor detection accuracy.

Similar to the regularly-shaped objects, the mean ESS for the artificial plants was slightly influenced by the sensor travel speed and was considerably influenced by the detection height. At sensor travel speeds of 1.6, 2.4, 3.2, 4.0, and 4.8 km h^−1^, the mean ESS was 0.59, 0.57, 0.55, 0.54, and 0.52 for the artificial plant 1 and was 0.64, 0.63, 0.61, 0.60, and 0.58 for the artificial plant 2, respectively. Instead, at the detection height of 0.25, 0.5, 0.75, and 1.0 m, the mean ESS was 0.51, 0.51, 0.58, and 0.61 for the artificial plant 1 and was 0.58, 0.59, 0.61, and 0.67 for the artificial plant 2. 

#### 3.2.6. Edge Detection of Objects at 0.25 m Apart

Additional edge detections for the objects arranged 0.25 m apart at 3.2 km h^−1^ travel speed and 0.5 m detection height showed the overall mean ESS for all the objects ranged from 0.95 to 0.58 as the horizontal distance increased from 0 to 3.5 m. Generally, the edge detection accuracy for the objects that were placed 0.25 m apart was higher than that of the objects arranged 0.5 m apart. The reason was that the laser sensor detected the objects from top to bottom, and the interference of the laser points reflected from the side surface of the objects became weaker when the objects placed closer to each other.

To maximize the space usage for optimal greenhouse production, plants are usually placed close to each other. This production practice benefits the application of laser sensors in greenhouses, because it is more accurate for the sensors to detect plants which are placed close to each other.

## 4. Summary

The accuracy of the 270° radial laser sensor to detect object shape and sizes was evaluated by the object edge similarity of paired images from the laser sensor and a digital camera. The edge detection accuracy was affected by the laser DR, which was significantly influenced by the sensor travel speed, and horizontal distance and sensor detection height. The DRS was inversely proportional to the sensor travel speed. High sensor speed produced negative effect on DRS. The influence on DRH coming from the horizontal distance at relatively lower sensor detection height was more significant than that at relatively greater detection height. These results revealed that relatively greater detection height could weaken the negative influence of the horizontal distances on DRH and improve DR.

The edge detection accuracy of the laser sensor was slightly influenced by the sensor travel speed, and significantly by the horizontal distance and sensor detection height. The ESS decreased slightly as the sensor speed increased from 1.6 to 4.8 km h^−1^. The ESS significantly decreased with increase of the horizontal distance in the range of 0 to 3.5 m. The detection results also showed that ESS increased as the sensor detection height increasing from 0.25 to 1.0 m in the test. Moreover, the influence from the horizontal distance of objects could be weakened by raising the sensor detection height in the range of 0.25 to 1.0 m. Based on the parameters evaluated in this research, the laser sensor setting of detection height of 1.0 m and travel speed of 1.6 km h^−1^ would optimize the laser sensor detection.

The detection results of the two sized artificial plants, as well as the rectangular box, demonstrated that the laser sensor could obtain a better performance in edge detection of those objects with flatter canopy surface.

Evaluation of objects with small interval spacing between two objects in the same row also illustrated that the edge detection accuracy became higher when the objects were placed closer together, which illustrated that the sensor was able to detect plants in greenhouse, because they were usually placed close to one another to maximize space usage for optimal greenhouse production.

In general, the laboratory test demonstrated the 270° radial laser scanning sensor was capable of detecting surface profiles of the complex-shaped objects at different horizontal distances and detection heights to the sensor with different sensor speeds. Future research will integrate this laser sensor and the control system to the variable-rate sprayers to automatically control the spray outputs based on plant structures and travel speeds in real time for greenhouse applications.

## Figures and Tables

**Table 1:** Test variables in the test for validating the laser sensor detection accuracy for object edge profiles.

Test Part	Variable								
1	Interval distance (m)	0.5							
	Speed (km h^−1^)	1.6	2.4		3.2		4.0		4.8
	Detection height (m)	0.25		0.50		0.75		1.00	
	Replication	3							
2	Interval distance (m)	0.25							
	Speed (km h^−1^)	3.2							
	Detection height (m)	0.5							
	Replication	3							

**Table 2:** Mean edge similarity score (ESS) of toy balls at five travel speeds (1.6, 2.4, 3.2, 4.0 and 4.8 km h^−1^) across eight horizontal distances and four different detection heights.

Speed (km h^−1^)	Horizontal Distance (m)							
	0	0.5	1.0	1.5	2.0	2.5	3.0	3.5
1.6	0.98	0.83	0.69	0.58	0.49	0.41	0.39	0.37
2.4	0.97	0.82	0.66	0.55	0.49	0.40	0.39	0.35
3.2	0.96	0.81	0.67	0.54	0.47	0.38	0.38	0.35
4.0	0.96	0.79	0.63	0.51	0.47	0.38	0.35	0.34
4.8	0.95	0.78	0.64	0.50	0.45	0.35	0.32	0.33

**Table 3:** Mean ESS of toy balls at four detection heights (0.25, 0.5, 0.75 and 1.0 m) across eight horizontal distances and five laser sensor travel speeds.

Detection Height (m)	Horizontal Distance (m)							
	0	0.5	1.0	1.5	2.0	2.5	3.0	3.5
0.25	0.96	0.64	0.54	0.37	0.36	0.36	0.33	0.29
0.5	0.96	0.74	0.61	0.43	0.36	0.33	0.34	0.30
0.75	0.97	0.91	0.73	0.66	0.56	0.37	0.35	0.35
1.0	0.96	0.93	0.76	0.69	0.62	0.48	0.44	0.46

**Table 4:** Mean ESS of four regularly-shaped objects (toy balls, basketballs, rectangular boxes and cylinders) and two sized artificial plants (plant 1 and plant 2) across all the combinations of five travel speeds (1.6, 2.4, 3.2, 4.0, and 4.8 km h^−1^) and four different detection heights (0.25, 0.5, 0.75 and 1.0 m) at eight horizontal distances.

Object	Horizontal Distance (m)							
	0	0.5	1.0	1.5	2.0	2.5	3.0	3.5
Toy ball	0.96	0.80	0.66	0.54	0.47	0.38	0.37	0.35
Basketball	0.95	0.79	0.68	0.58	0.49	0.41	0.38	0.38
Rectangular box	0.97	0.90	0.84	0.73	0.64	0.54	0.49	0.48
Cylinder	0.96	0.85	0.69	0.61	0.51	0.37	0.33	0.31
Plant 1	0.90	0.72	0.67	0.61	0.45	0.37	0.35	0.35
Plant 2	0.94	0.81	0.78	0.64	0.48	0.44	0.41	0.38

**Table 5:** Mean ESS of basketballs across four different detection heights (0.25, 0.5, 0.75 and 1.0 m) at eight horizontal distances and five travel speeds.

Speed (km h^−1^)	Horizontal Distance (m)							
	0	0.5	1.0	1.5	2.0	2.5	3.0	3.5
1.6	0.96	0.81	0.72	0.63	0.52	0.43	0.40	0.40
2.4	0.95	0.80	0.69	0.60	0.52	0.42	0.38	0.37
3.2	0.96	0.79	0.68	0.58	0.48	0.40	0.37	0.37
4.0	0.94	0.79	0.65	0.54	0.50	0.40	0.35	0.35
4.8	0.95	0.77	0.66	0.53	0.45	0.39	0.34	0.36

**Table 6:** Mean ESS of basketballs across five travel speeds (1.6, 2.4, 3.2, 4.0 and 4.8 km h^−1^) at eight horizontal distances and four different detection heights.

Detection Height (m)	Horizontal Distance (m)							
	0	0.5	1.0	1.5	2.0	2.5	3.0	3.5
0.25	0.95	0.61	0.53	0.41	0.38	0.30	0.34	0.36
0.5	0.95	0.71	0.64	0.50	0.43	0.35	0.35	0.35
0.75	0.96	0.91	0.72	0.64	0.50	0.38	0.36	0.39
1.0	0.95	0.94	0.84	0.75	0.66	0.61	0.45	0.42

**Table 7:** Mean ESS of rectangular boxes across four different detection heights (0.25, 0.5, 0.75 and 1.0 m) at eight horizontal distances and five sensor travel speeds.

Speed (km h^−1^)	Horizontal Distance (m)							
	0	0.5	1.0	1.5	2.0	2.5	3.0	3.5
1.6	0.98	0.92	0.86	0.79	0.68	0.60	0.50	0.51
2.4	0.97	0.91	0.85	0.73	0.64	0.55	0.50	0.48
3.2	0.97	0.90	0.84	0.73	0.64	0.53	0.49	0.48
4.0	0.97	0.90	0.84	0.71	0.62	0.51	0.48	0.47
4.8	0.96	0.90	0.81	0.70	0.61	0.50	0.45	0.46

**Table 8:** Mean ESS of rectangular boxes across five travel speeds (1.6, 2.4, 3.2, 4.0 and 4.8 km h^−1^) at eight horizontal distances and four detection heights.

Detection Height (m)	Horizontal Distance (m)							
	0	0.5	1.0	1.5	2.0	2.5	3.0	3.5
0.25	0.98	0.88	0.82	0.71	0.60	0.54	0.42	0.41
0.5	0.98	0.91	0.82	0.72	0.64	0.49	0.44	0.43
0.75	0.97	0.91	0.84	0.73	0.65	0.56	0.52	0.53
1.0	0.96	0.92	0.87	0.75	0.66	0.57	0.55	0.54

**Table 9:** Mean ESS of cylinders across four different detection heights (0.25, 0.5, 0.75 and 1.0 m) at eight horizontal distances and five travel speeds.

Speed (km h^−1^)	Horizontal Distance (m)							
	0	0.5	1.0	1.5	2.0	2.5	3.0	3.5
1.6	0.97	0.86	0.74	0.66	0.55	0.39	0.35	0.33
2.4	0.95	0.85	0.72	0.63	0.54	0.39	0.33	0.32
3.2	0.96	0.86	0.68	0.60	0.51	0.37	0.33	0.31
4.0	0.94	0.85	0.66	0.59	0.49	0.36	0.33	0.29
4.8	0.95	0.85	0.65	0.58	0.46	0.35	0.31	0.30

**Table 10:** Mean ESS of cylinders across five travel speeds (1.6, 2.4, 3.2, 4.0 and 4.8 km h^−1^) at eight horizontal distances and four detection heights.

Detection Height (m)	Horizontal Distance (m)							
	0	0.5	1.0	1.5	2.0	2.5	3.0	3.5
0.25	0.96	0.83	0.58	0.56	0.43	0.30	0.26	0.24
0.5	0.95	0.83	0.63	0.56	0.47	0.30	0.23	0.22
0.75	0.96	0.86	0.73	0.61	0.55	0.43	0.38	0.37
1.0	0.95	0.90	0.81	0.73	0.58	0.47	0.44	0.41

**Table 11:** Mean ESS of artificial plant 1 across four detection heights (0.25, 0.5, 0.75 and 1.0 m) at eight horizontal distances and the five travel speeds.

Speed (km h^−1^)	Horizontal Distance (m)							
	0	0.5	1.0	1.5	2.0	2.5	3.0	3.5
1.6	0.92	0.74	0.70	0.64	0.50	0.42	0.39	0.37
2.4	0.91	0.73	0.69	0.63	0.47	0.42	0.37	0.37
3.2	0.90	0.73	0.67	0.61	0.46	0.36	0.35	0.35
4.0	0.89	0.71	0.66	0.60	0.41	0.32	0.33	0.34
4.8	0.88	0.71	0.64	0.57	0.40	0.32	0.32	0.31

**Table 12:** Mean ESS of artificial plant 1 across five travel speeds (1.6, 2.4, 3.2, 4.0 and 4.8 km h^−1^) at eight horizontal distances and four detection heights.

Detection Height (m)	Horizontal Distance (m)							
	0	0.5	1.0	1.5	2.0	2.5	3.0	3.5
0.25	0.91	0.69	0.66	0.60	0.38	0.26	0.27	0.29
0.5	0.89	0.74	0.65	0.60	0.40	0.27	0.27	0.27
0.75	0.91	0.73	0.66	0.61	0.45	0.46	0.42	0.40
1.0	0.89	0.73	0.71	0.64	0.56	0.47	0.45	0.44

**Table 13:** Mean ESS of plant 2 across four detection heights (0.25, 0.5, 0.75, and 1.0 m) at eight horizontal distances (0, 0.5, 1.0, 1.5, 2.0, 2.5, 3.0, and 3.5 m) and five travel speeds (1.6, 2.4, 3.2, 4.0, and 4.8 km h^−1^).

Speed (km h^−1^)	Horizontal Distance (m)							
	0	0.5	1.0	1.5	2.0	2.5	3.0	3.5
1.6	0.94	0.83	0.80	0.68	0.54	0.49	0.45	0.42
2.4	0.94	0.82	0.79	0.67	0.52	0.45	0.43	0.41
3.2	0.94	0.81	0.80	0.63	0.47	0.45	0.40	0.37
4.0	0.94	0.80	0.76	0.63	0.44	0.43	0.39	0.36
4.8	0.93	0.82	0.74	0.61	0.41	0.40	0.37	0.36

**Table 14:** Mean ESS of plant 2 at eight horizontal distances (0, 0.5, 1.0, 1.5, 2.0, 2.5, 3.0, and 3.5 m) across five travel speeds (1.6, 2.4, 3.2, 4.0, and 4.8 km h^−1^) at four different detection heights (0.25, 0.5, 0.75, and 1.0 m).

Detection Height (m)	Horizontal Distance (m)							
	0	0.5	1.0	1.5	2.0	2.5	3.0	3.5
0.25	0.94	0.74	0.77	0.65	0.43	0.41	0.35	0.33
0.5	0.94	0.83	0.79	0.65	0.45	0.43	0.35	0.29
0.75	0.94	0.84	0.81	0.63	0.46	0.41	0.36	0.41
1.0	0.94	0.85	0.74	0.65	0.56	0.53	0.58	0.51
